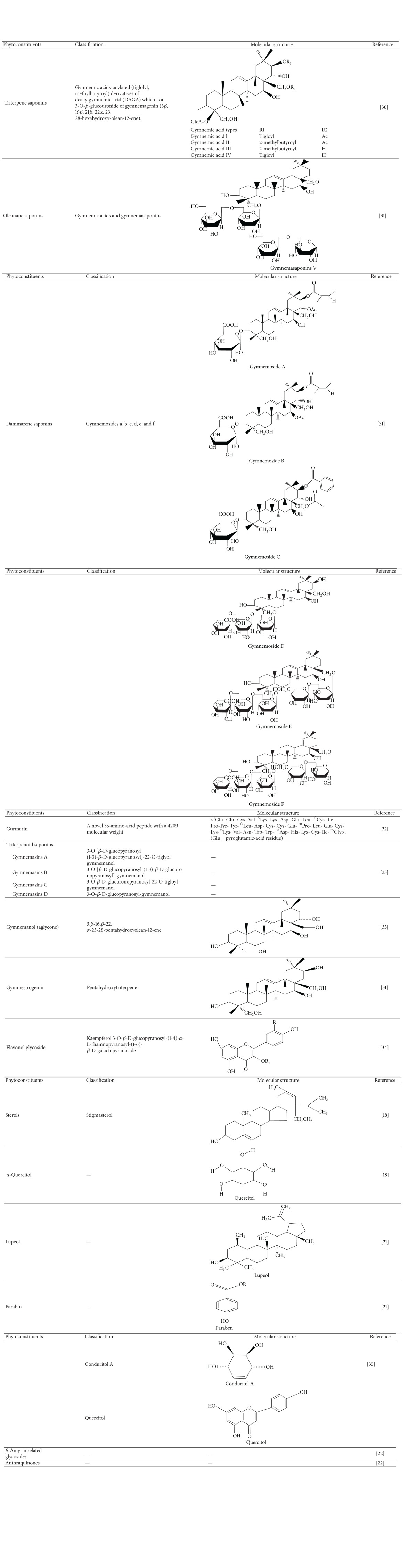# Phytochemical and Pharmacological Properties of *Gymnema sylvestre*: An Important Medicinal Plant

**DOI:** 10.1155/2014/830285

**Published:** 2014-01-06

**Authors:** Pragya Tiwari, B. N. Mishra, Neelam S. Sangwan

**Affiliations:** ^1^Metabolic and Structural Biology Department, Central Institute of Medicinal and Aromatic Plants (CSIR-CIMAP), P.O. CIMAP, Lucknow 226015, India; ^2^Department of Biotechnology, Gautam Buddh Technical University, Lucknow, Uttar Pradesh 226021, India

## Abstract

*Gymnema sylvestre* (Asclepiadaceae), popularly known as “gurmar” for its distinct property as sugar destroyer, is a reputed herb in the Ayurvedic system of medicine. The phytoconstituents responsible for sweet suppression activity includes triterpene saponins known as gymnemic acids, gymnemasaponins, and a polypeptide, gurmarin. The herb exhibits a broad range of therapeutic effects as an effective natural remedy for diabetes, besides being used for arthritis, diuretic, anemia, osteoporosis, hypercholesterolemia, cardiopathy, asthma, constipation, microbial infections, indigestion, and anti-inflammatory. *G. sylvestre* has good prospects in the treatment of diabetes as it shows positive effects on blood sugar homeostasis, controls sugar cravings, and promotes regeneration of pancreas. The herbal extract is used in dietary supplements since it reduces body weight, blood cholesterol, and triglyceride levels and holds great prospects in dietary as well as pharmacological applications. This review explores the transition of a traditional therapeutic to a modern contemporary medication with an overview of phytochemistry and pharmacological activities of the herb and its phytoconstituents.

## 1. Introduction

The naturopathic treatment for diseases has been explored extensively since ancient times and gaining momentum in the present scenario. Indian flora accounts for about 45,000 plant species out of which several thousands have pharmacological significance [1]. Diabetes mellitus is a major endocrine disorder affecting nearly 10% of the population worldwide [2] and a key issue of concern. The disease in its severe state affects major systems of the body, leading to multiorgan complications. Oral hypoglycemic agents like sulphonylureas and biguanides are the conventional drugs used for the treatment, but the adverse side effect associated with these drugs is a major limitation. The herbal medicines are becoming popular due to better results and safe use as compared to marketed drugs and more effective treatment of health problems [3]. Plants possessing antidiabetic activities are of significant interest for ethnobotanical community as they are recognized to contain valuable medicinal properties in different parts and a number of them have shown varying degree of hypoglycemic and antihyperglycemic activity [1]. The bioactive constituents found in many plant species are isolated for direct use as drugs, lead compounds, or pharmacological agents. These traditional approaches might offer a natural key to unlock diabetic complications [4]. The chemical structures of a phytomolecule play a critical role in its antidiabetic activity. Several plant species being a major source of terpenoids, flavonoids, phenolics, coumarins, and other bioactive constituents have shown reduction in blood glucose levels [5, 6]. Various antidiabetic plant extracts like aloe (*Aloe vera L*), bitter Melon (*Momordica charantia*), fenugreek (*Trigonella foenum-graecum*), Asian ginseng (*Panax ginseng C.A.Meyer*) and American ginseng (*Panax quinquefolius L*), gymnema (*Gymnema sylvestre*), milk thistle (*Silybum marianum*), nopal (*Opuntia streptacantha*), salacia (*Salacia oblonga; Salacia Reticulate*), and formulations like those of chromium have been used and clinically tested for their activity as well as potential side effect [7].

The present review is a research update on *Gymnema sylvestre*, a rare herb with significant medicinal attributes with an overview of its ethnobotanical uses, phytochemistry dealing with an in-depth study of its phytochemicals, and their bioactivities. It also explores the facts and prospects of its development into a modern and efficient therapeutic, contemporary with the present trends of pharmacology and drug development. Furthermore, it holds significant prospects in major health problems like cardiovascular disorders, obesity, osteoporosis, and asthma besides being a popular medication for number of other health ailments. The herb finds significant application in various food preparations for control of obesity and blood cholesterol levels besides regulation of sugar homeostasis. The herbal preparations of *G. sylvestre* are presently used in tea bags, health tablets and supplements, beverages, and confectioneries.

## 2. Traditional Perspective

*G. sylvestre* is an indigenous herb, belonging to the class dicotyledonous of the family Asclepiadaceae. The plant is a good source of a large number of bioactive substances [8]. It has deep roots in history, being one of the major botanicals used in Ayurvedic system of medicine to treat conditions ranging from diabetes, malaria, to snakebites [9]. The herb is cultivated worldwide and also known as Chigengteng or Australian Cowplant, *Waldschlinge* in German, periploca of the woods in English and gurmar in Hindi [10].

## 3. Taxonomy

*G. sylvestre R.Br. *is a perennial, woody climber belonging to family Asclepiadaceae or the “milk weed” family [11]. The genus is classified into 40 species, some of which like *G. sylvestre*, *G. montanum*, *G. yunnanense*, and *G. inodorum* have medicinal properties [12–14]. The plant is found in tropical and subtropical regions, well distributed in parts of central and southern India and in the southern part of China, tropical Africa, Malaysia, and Sri Lanka [9]. *G. sylvestre* is slow growing herb, found ideally in tropical and subtropical humid climate and common in hills of evergreen forests. It is a climber and generally requires support for growth. The seeds are sown in the months of November-December and harvested from September to February. The propagation through seed germination is difficult due to low viability of the seeds; therefore, the alternative has been root cuttings which are generally planted in the months of June and July [15]. Terminal cuttings with three of four nodes have also been used as for vegetative propagation and usually planted in the month of February-March [16]. The leaves are opposite, usually elliptic or ovate (1.25–2.0 inch × 0.5–1.25 inch), inflorescence is lateral umbel in cymes; follicles are terete and lanceolate, up to 3 inches in height. Corolla is pale yellow in colour, valvate, campanulate with single corona with 5 fleshy scales. The calyx-lobes are long, ovate, obtuse, and pubescent. Carpels-2, unilocular, ovules locules may be present, anther connective produced into a membranous tip [17, 18].

## 4. Phytochemical Profiling

The leaves of *G. sylvestre *contain triterpene saponins belonging to oleanane and dammarane classes. The major constituents like gymnemic acids and gymnemasaponins are members of oleanane type of saponins while gymnemasides are dammarane saponins [19, 20]. Other phytoconstituents include anthraquinones, flavones, hentriacontane, pentatriacontane, phytin, resins, tartaric acid, formic acid, butyric acid, lupeol, *β*-amyrin related glycosides, stigmasterol, and calcium oxalate [21]. The presence of alkaloids had been detected in plant extracts. Leaves of *G. sylvestre* have acidic glycosides and anthraquinones and their derivatives [22]. The major secondary metabolites in *Gymnema* includes a group of nine closely related acidic glycosides, the main are gymnemic acid A–D and found in all parts of the plant (see Supplementary Table 1 in supplementary materials available online at http://dx.doi.org/10.1155/2014/830285). The maximum content of gymnemic acid is found in shoot tips (54.29 mg-g^−1^ DW) and least in seeds (1.31 mg-g^−1^ DW). Antisaccharin property of gymnemic acid A_1_ was greatly reduced on conversion into A_2_, while no activity was observed in case of A_3_ suggesting that the ester group in the genin portion of gymnemic acid imparts the antisweet property to the triterpene saponins, the gymnemic acids. Gymnemic acids A_2_ and A_3_ possessed both glucuronic acid and galactose in their molecular structures while glucuronic acid was found to be the only moiety in gymnemic acid A_1_ [23]. Further, a series of gymnemic acids (gymnemic acid I, II, III, IV, V, VI, and VII) were isolated and characterized from the hot water extract of dry leaves of *G. sylvestre* [24, 25]. The Gymnemic acids comprise of several members designated as gymnemic acids I–VII, gymnemosides A–F, and gymnemasaponins Table 1. The derivatives of gymnemic acids are several acylated tigloyl, methylbutyryl group substituted members, derived from deacylgymnemic acid (DAGA) which is a 3-O-*β*-glucuronide of gymnemagenin (3*β*, 16*β*, 21*β*, 22*α*, 23, 28-hexahydroxy-olean-12-ene). Gymnemic acid A comprises of gymnemic acids A_1_, A_2_, A_3_, and A_4_ and named gymnemagenin. This constituent is a D-glucuronide of hexahydroxy-triterpene that esterifies with acids [26]. Other five gymnemic acids, namely, VIII, IX, X, XI, and XII, were isolated and characterized later [27]. Gymnemasaponins III, another antisweet compound, isolated from *G. sylvestre* was found to consist of 23 hydroxylongispinogenin as the aglycone moiety glycosylated with either one or two glucose molecules at both the 23 or 28 hydroxyl groups [28]. These compounds exhibited lesser antisweet effect than those of gymnemic acids [29].

Gurmarin, an important 35 amino-acid peptide having a molecular weight of 4209, was isolated from *G. sylvestre *[32]. The sugar suppression activity of this compound was determined electrophysiologically on the taste responses of rat [36]. The antisweet effect of this polypeptide is very specific to sweet taste on tongue, affected by the pH change. It has been reported that the polypeptide exhibited maximum antisweetner property near its isoelectric point [37]. The hydrophobic, rather than the ionic, interaction plays a significant role in proper binding of gurmarin to the target molecules [32, 38]. The other important constituents isolated from leaves are gymnemasins A, B, C, and D and alkaloids [39]. A number of saponins such as gymnemic acid, deacyl gymnemic acid, gymnemagenin [40], 23-hydroxylnogispinogenin, and gymnestrogenin have been purified [33, 41, 42] from *G. sylvestre*. The phytochemicals in leaf extract were also analyzed through gas chromatography coupled to mass spectrometry and identified for the presence of terpenoids, glycosides, saturated and unsaturated fatty acids, and alkaloids in three different leaves extract, namely, petroleum ether, chloroform, and methanol as solvents used for extraction [43]. The bioactive constituents present in the plant were found to be mixture of diverse phytomolecules such as gymnemic acids, gymnemosides, gymnemasaponins, gurmarin, gymnemanol, stigmasterol, d-quercitol, *β*-amyrin related glycosides, anthraquinones, lupeol, hydroxycinnamic acids, and coumarols group.

## 5. Biosynthesis and Genomics

Saponins, natural products widespread in plant kingdom, are glycosides composed of triterpenoids or steroidal aglycones moieties [44] and the aglycones are known as sapogenins. Many plant-derived saponins, namely, ginsenosides, soyasaponins, and saikosaponins have been found to exhibit significant anticancer activity. Besides, some saponins display pharmacological properties, namely, anticholesterolemic, adjuvant hemolytic, and anticancer [45–47]. It was also found that the foods originating from plants having an increased level of triterpenes are thought to have a cholesterol lowering effect. Transgenics with altered levels of triterpenes may be resistant to pests and increased saponin content will confer enhanced nutritional value to the plant.

Triterpenoid saponins are a class of plant secondary metabolites originated via the isoprenoid pathway by cyclization of 2,3-oxidosqualene precursor in which one or more sugar residues are added [48] and leading to the formation of the triterpenoid skeleton of b-amyrin and related glycosides. The presence of polar nucleus, linked to one or more sugar residues, is responsible for the characteristic activities of these compounds [44]. Majority of the significant steps at molecular level in triterpene saponin biosynthesis remain uncharacterized. The steps involving the biosynthesis of b-amyrin by b-amyrin synthase, an oxidocyclase, have been well characterized in several plant species including *Arabidopsis thaliana* [49], oat [50], but steps involving the modification of the triterpenoid backbone by the cytochrome P450-dependent monooxygenases and uridine diphosphate glycosyltransferases remain less understood.

Extensive research has gone into the metabolic profiling of* G. sylvestre*, but there are very few reports pertaining to metabolomics and genomics. The structural elucidation of gymnemic acid revealed the presence of triterpene aglycone moiety known as sapogenin attached to a sugar chain. The occurrence of significant percentage of triterpene glycosides in plant indicates that glycosylation is a critical process in the modification/generation of triterpene saponins. Studies including the metabolomics and functional genomics with emphasis on the gene identification, cloning, and their functional characterization will be an important tool in deciphering the functional role of these genes in the biochemical pathway leading to medicinal properties of the phytoconstituents in the plant.

**Figure 1:**
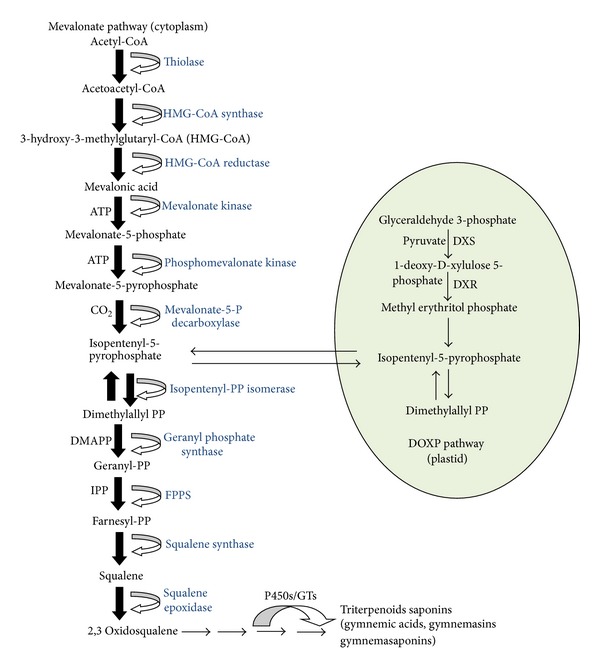
Hypothetical pathway of Gymnemic acid biosynthesis. The general sketch represents the formation of triterpenoids through Mevalonate pathway. Further, it was assumed that gymnemagenin (sapogenin) gave rise to gymnemic acids and derivatives through glycosylation mechanism by glycosyltransferases.

Further, in an attempt to understand the molecular mechanism of genes responsible for medicinal properties of *G. sylvestre*, two partial cds (accession nos. GU191124; GU181368) were submitted to NCBI database [51, 52]. Further studies into the identification and characterization of genes involved in the biosynthesis of triterpene glycosides, gymnemic acids will provide valuable information in deciphering the biosynthetic pathway of gymnemic acids and the mechanism of their pharmacological activities in the plant. Since, the transcriptome data of *Gymnema sylvestre* is unavailable and various proteins and enzymes at the biochemical level remain uncharacterized, so the exact mechanism of Gymnemic acid biosynthesis is not reported in the literature. However, extensive research is ongoing in our lab to decode the functional role of glycosyltransferases in biosynthesis of Gymnemic acids owing to its significant pharmacological importance (unpublished data). The biosynthesis pathway of gymnemic acid remains unknown; however, putatively pathway for triterpene glycosides is derived from the isoprenoid pathway with glycosylation of the triterpene aglycone at the terminal transformation of gymnemagenin. A general diagrammatic sketch has been drawn to represent a putative pathway with a focus on terminal pathway steps in biosynthesis of saponins from *Gymnema sylvestre* (Figure 1).

## 6. Mechanism of Action of Gymnemic Acids

The mode of action of the drug is through stimulation in insulin secretion from pancreas [53]. It also exerts a similar effect by delaying the glucose absorption in the blood. The atomic arrangements of gymnemic acids to the taste buds are similar to sugar molecules which fill the receptors in the taste buds preventing its activation by the sugar molecule in the food. Similarly, in the intestine it attaches to the receptor present in external layer of intestine, thereby preventing the absorption of sugar molecules by intestine, leading to reduction in blood sugar levels [33]. Gurmarin acts in a similar manner by interfering with the ability of taste buds on the tongue to differentiate between sweet and bitter. Hypoglycemic effect of gymnemic acids includes a cascade of events starting from modulation of incretin activity which triggers insulin secretion and release. It also increases regeneration of pancreatic islet cells to enhanced enzyme mediated uptake of glucose. This process decreased glucose and fatty acid assimilation in the small intestine and interferes in the ability of receptors in mouth and intestine to sensation of sweetness. It has been previously reported in the literature that the action of gymnemic acid is similar to that of incretin-mimetic mechanism of action [54]. Gymnemic acid has been found to interact with glyceraldehyde-3-phosphate dehydrogenase (GAPDH), a key enzyme in glycolysis pathway [55]. The findings also indicated that the acyl moieties present in gymnemic acids play important role for the GA-induced smearing of GAPDH and G3PDH and play an integral role in the antihyperglycemic activity of GA derivatives [56].

## 7. Pharmacological Activities of Extracts and Pure Compounds Isolated from *Gymnema sylvestre*

Although the herb is widely used as a naturopathic treatment for diabetes [57, 58], it also demonstrates promising effects in the treatment of obesity, arthritis, hyperlipidemia, Parkinsonism, and hypercholesterolemia [59–61]. Furthermore, the bioactive compounds of plant have antimicrobial, anti-inflammatory, and anticancer properties. The leaves of the plant are used for the treatment of obesity [62], dental caries [63], antibiotic, in stomachache, blood purifier, and in rheumatism [64]. Some of the significant pharmacological properties of the herb had been discussed in detail. Various plant parts, namely, leaves, roots possess medicinal properties and used for the treatment of various diseases in Ayurvedic system of medicine (supplementary Table 2). Numerous bioactive compounds isolated from the plant either as pure compounds or as crude extracts possess medicinal properties and clinically tested in animal model systems for scientific validation (supplementary Table 3).

### 7.1. Antidiabetic Property

The herb accounts for its sweet inactivation property to the presence of triterpene saponins known as gymnemic acids, gymnemasaponins, and gurmarin. Experimental trials confirmed the hypoglycemic effect of *G. sylvestre* on beryllium nitrate and streptozotocin treated rats. There was a slight increase in body weight and protein and a significant decrease in fasting blood glucose in diabetic rats treated with *G. sylvestre*, *C. auriculata*, *E. jambolanum*, and *S. reticulata *and the effects were quite similar to insulin and glibenclamide treated mice.

An investigation to determine the antioxidant activity of *Gymnema *leaf extract and the role of antioxidants in diabetic rats was performed by Kang et al. [65] using ethanolic extracts. Several antioxidant assays, namely, thiobarbituric acid (TBA) assay with slight modifications, using egg yolk lecithin or 2-deoxyribose (associated with lipid peroxidation), superoxide dismutase- (SOD-) like activity assay, and 2,2′-Azinobis (3-ethylbenzothiazoline-6-sulfonic acid) (ABTS) assay (involved in electron or radical scavenging), depicted significant antioxidant activity of the ethanolic extract. Further LC/MS analysis revealed the presence of antihyperglycemic compounds like gymnemagenin and gymnemic acids in *G. sylvestre *extract and the level of lipid peroxidation reduced by 31.7% in serum, 9.9% in liver, and 9.1% in kidney in diabetic rats fed with the ethanolic extract. The activity of transaminases in gluconeogenesis and ketogenesis in diabetes like glutamate pyruvate transaminase (GPT) in serum and glutathione peroxidase in cytosolic liver returned to normal levels after the administration of ethanolic leaf extract in diabetic rats [66]. Antihyperglycemic effect of crude saponin fraction and five triterpene glycosides (gymnemic acids I–IV and gymnemasaponin V), isolated from the methanolic extract of the leaves, was reported [56]. It was found that gymnemic acid IV (3.4/13.4 mg/kg) decreased blood glucose levels by 14.0–60.0% within 6 hours of administration as compared to glibenclamide. It has been reported that gymnemic acid IV increased plasma insulin levels in STZ-diabetic mice at a concentration of 13.4 mg/kg while it did not cause inhibitory effect on *α*-glucosidase activity in the brush border membrane vesicles of small intestine in normal rat.

Similarly, in an experimental study, the antidiabetic and hypolipidemic potential of dried powdered leaves of *G. sylvestre* was investigated. The effect of *G. sylvestre* leaf extract was administered to nondiabetic and alloxan-diabetic rats. It was found that the *Gymnema* leaf extract had no effect on the alleviated glycemia caused by balanced meal or due to the administration of glucose or amylose but increased serum lipid level after SOC treatment. However, in nondiabetic and alloxan diabetic rats the subacute and chronic treatment with *Gymnema* extract had no effect on the ingestion of food and water, gain of body weight, and the level of glucose and lipid in blood. But the herbal formulation requires clinical approval and scientific validation before being used for the treatment of diabetes and hyperlipidemia [67]. Finally, it was concluded from the studies that the herb possesses antidiabetic effect and sugar inactivation properties.

### 7.2. Antiarthritic Activity

The leaf extract of *G. sylvestre* was examined for antiarthritic activity on albino rats. The water soluble and petroleum ether (40–60°C) extract was found to be significantly effective in controlling arthritis. It was also assumed that the most potent antiarthritic activity of the leaves may be due to the nature of triterpenoids, steroids, and saponin glycosides [59]. Different extracts were suspended with 1% Tween 80, and the drug Diclofenac sodium was administered once daily through oral route and the effect was monitored for 21 days. It was observed that the rats developed swelling in multiple joints on induction with an adjuvant and exhibited inflammation in cells, bone destruction, and reshaping. The petroleum ether extract treated group showed significant reduction in paw swelling possibly due to inhibiting the response of inflammatory cells or blocking the release of mediators like cytokines (IL-Ib and TNF-a), GM-CSF, interferons, and PGDF which are responsible for pain and disabilities arising due to destruction of bone and cartilage [68]. The other possible mechanism of action suggested protection of the release of joint cartilage and bone destruction in chronic arthritic model [59]. The multiple studies employing use of polar solvents in extract preparations by investigators demonstrated the antiarthritic potential of the leaf extract.

### 7.3. Treatment of Dental Caries

Dental caries can be defined as infection of tooth, occurring due to various kinds of gram-positive cariogenic bacteria [69] like *S. aureus, S. mitis*, and* S. mutans, *and fungus-like *Candida albicans *which attaches to the tooth surface through release of extracellular polysaccharides from sucrose and metabolize sugar to organic acid mainly lactic acid resulting in demineralization of the tooth enamel [70]. The chloroform, petroleum ether, and methanolic leaf extracts of *G. sylvestre* at various concentrations of 25, 50, and 100 mg/mL were tested against microbial dental infections and found to be significantly effective against these cariogenic bacteria particularly the methanolic extract which showed highest activity at minimum concentration. The good potential of the hydroalcoholic extract of the plant leads to the development and manufacture of gurmar tooth powdered marketed as “Gurmar Herbal tooth paste” and “Gurmar Herbal Tooth powder.” These herbal formulations offer new prospects in the treatment of dental caries once clinically approved by the scientific community [63].

### 7.4. Antibiotic and Antimicrobial Activity

The antibiotic and antimicrobial activity of different extracts of *G. sylvestre* was determined [71] against a number of pathogens, namely, *S. aureus*, *E. coli*,* and B. subtilis *while no activity was observed against gram-negative bacteria. *G. sylvestre* leaf extracts showed good prospects as an antibiotic herbal remedy was effective as herbal formulation for the treatment of microbe's related infections [71]. The antibacterial activity of *G. sylvestre *and gymnemic acid was also studied against *E. coli *and* B. cereus* and the antimicrobial effect was significant against the microbes [72]. Bhuvaneswari et al. [73] demonstrated that the methanolic extracts of* G. sylvestre* were assessed for antimicrobial activity of aerial and root parts separately. The result exhibited that the methanol extracts in acidic range have good activity towards all the pathogens showing its broad spectrum nature. In a similar study, the antimicrobial effect of ethanolic extract of* G. sylvestre *against *Bacillus pumilus*, *B. subtilis*, *P. aeruginosa*, and *S. aureus* showed promising antimicrobial effect [74]. It can be inferred from the studies that the methanolic and ethanolic leaf extract of *Gymnema sylvestre* possesses considerable antibiotic and antimicrobial activity.

### 7.5. Anti-Inflammatory Activity

In the Ayurvedic system of medicine, the leaf of *G. sylvestre *has been widely used and is considered as bitter, acrid, thermogenic, digestive, liver tonic, anodyne, and anti-inflammatory [75]. The bioactive constituents in *G. sylvestre* known as tannins and saponins are responsible for the anti-inflammatory activity of the plant [76]. In the study, carrageenin induced paw oedema and cotton pellet induced granuloma rats were taken, and the aqueous extract of *G. sylvestre* leaf was investigated for its anti-inflammatory activity at the doses of 200, 300, and 500 mg/kg with drug, phenylbutazone as standard. It was found that the gymnema aqueous extract at a concentration of 300 mg/kg significantly decreased the paw oedema volume by 48.5% within 4 hours of administration while the drug phenylbutazone decreased the paw oedema volume by 57.6%. Also, the aqueous extract at a concentration of 200 and 300 mg/kg exhibited reduction in granuloma when compared with the control group [77].

### 7.6. Anticancer and Cytotoxic Activity

Many plant-derived saponins, namely, ginsenosides, soyasaponins, and saikosaponins have been found to exhibit significant anticancer activity. Anticancer potential of gymnemagenol on *HeLa *cancer cell lines in *in vitro* conditions, was determined [78]. The cytotoxic activity of the saponins was tested by MTT cell proliferation assay. Different concentrations of gymnemagenol (5, 15, 25, and 50 *μ*g/mL) were taken and plates were incubated for 48 hours. The IC_50_ value was found to be 37 *μ*g/mL for gymnemagenol and after 96 hours, the extract at a concentration of 50 *μ*g/mL showed good cytotoxic activity on 73% on* HeLa* cells. The isolated bioactive constituent, gymnemagenol, was found to show a high degree of inhibition to the proliferation of *HeLa* cancer cell line. Further, these saponins were not toxic to the growth of normal cells under *in vitro* conditions [79]. With the rising percentage of cancer in people, the herbal formulation is a prospective medication in cancer therapy.

### 7.7. Antihyperlipidemic Activity

The prevalence of coronary artery disease is the cause of higher incidence of mortality than other causes combined [80]. The major factor contributing to atherosclerosis and related disorders like coronary artery diseases is hyperlipidemia [81]. Reduction in serum cholesterol levels may significantly reduce the chances of coronary heart disease [80]. Due to the limitations of synthetic drugs in having adverse effects, plant-based formulations offer a good prospect for the treatment of heart disease. Gymnemic acids preparations have been found to be effective against obesity [42]. The triterpene saponins constitute several acylated (tigloyl, methylbutyryl, etc.) derivatives of deacylgymnemic acid. Gymnemic acids consist of gymnemic acids I–VII, gymnemosides A–F, gymnemasaponins, and so forth [17]. In the study, high cholesterol diet, standard atorvastatin, and high cholesterol diet with hydroalcoholic extract of gymnemic acid were fed to female rats for seven days. It was observed that the rats fed with high cholesterol diet showed increase in serum cholesterol, serum triglycerides, low-density lipoprotein cholesterol, and very low-density lipoprotein and significant decrease in high-density lipoprotein cholesterol in comparison to normal animals. The group administered with hydroalcoholic extract of *Gymnema* leaves at a dose of 200 mg/kg showed significant reduction in the levels of all lipids with increase in HDL-C as compared to high cholesterol diet control [60]. A study demonstrated that the hexane extract of the leaves of *G. sylvestre* possesses antiobesity activity. It was found that, after 45 days of administration of hexane extract of *G. sylvestre*, a significant reduction in increased body weight and high temperature due to obesity was observed. Also, the hexane extract improved the cholesterol, triglyceride, LDL, and HDL levels. The hexane extract of the leaves of *G. sylvestre* have the potential to treat obesity comparable with that of standard drug, atorvastatin [82]. The studies showed that the leaf extract has good prospects in the reduction of cholesterol levels and as a herbal medication for obesity.

### 7.8. Immunostimulatory Activity

Immunomodulation is referred to as the regulation or control of the immunity which involves the enhancement or reduction in the immune responses. The body response to a particular condition might be regulated by agent that enhances or suppresses its action [83]. *G. sylvestre* is reported to be an immunostimulatory plant and the leaves possess immunostimulatory effect [84]. The aqueous leaf extract was tested for immunostimulatory activities by detecting the movement of neutrophils, chemotaxis tests, phagocytosis of killed *C. albicans*, and nitroblue tetrazolium assays. Aqueous leaf extract of *G. sylvestre* showed remarkable immunostimulatory activity at 10, 25, 50, 100, and 1000 *μ*g/mL on human neutrophils under* in vitro* conditions [85].

### 7.9. Hepatoprotective Activity

The hepatoprotective effect of hydro-alcoholic extract of *G. sylvestre *was evaluated by Srividya et al. [86]. The rat hepatocytes (freshly prepared) were subject to treatment with different concentration of hydroalcoholic extract prepared by the hot maceration process. The extract at a concentrations of 200, 400, and 600 *μ*g/mL showed significant antihepatotoxicity against the D-galactosamine-induced hepatotoxicity, and the concentration of 800 *μ*g/mL was found to be cytotoxic. The cells exhibited a significant restoration of the altered biochemical parameters towards the normal (*P* < 0.001) when compared to D-galactosamine treated groups in a dose-dependent manner, when treated with the hydroalcoholic extract different extracts of *G. sylvestre*.

### 7.10. Wound Healing Activity

The alcoholic extract of leaves of *G. sylvestre* was found to exhibit significant wound healing activity in rats [85]. According to Kiranmai et al. [87], hydroalcoholic extract of *G. sylvestre *has good wound healing property as compared with control group. TLC analysis, wound contraction, and qualitative tests supported the synergistic wound healing effect of the plant. The increased wound healing activity of hydroalcoholic extracts may be attributed to the free radical scavenging action and the presence of phytoconstituents (flavonoids) which may act individually or have additive effect. The flavonoids in alcoholic extract were detected by TLC and phytochemical analysis [88].

### 7.11. Ethnobotanical**  **Uses

Traditionally, the leaves of *G. sylvestre* were used for the treatment of diabetes and other disorders, while the flowers and bark are given in diseases related to phlegm [89]. The ancient literature on Indian medicine, *Sushruta*, describes gurmar as a destroyer of madhumeha (glycosuria) and other urinary disorders. The extract of *G. sylvestre* is reported to be a bitter acrid, anti-inflammatory, anodyne, digestive, liver tonic, emetic, diuretic, thermogenic, stomachic, stimulant, anthelmintics, laxative, cardiotonic, expectorant, antipyretic, and uterine tonic. The plant also exhibits medicinal importance in the treatment of jaundice, constipation, cardiopathy, asthma, bronchitis, amenorrhoea, conjunctivitis, renal and vesical calculi, dyspepsia, leucoderma, and Parkinsonism [90]. Reports in the ancient literature suggested that the plant has multiple medicinal applications, namely, antihelminthic, antipyretic, astringent, an alexipharmic, anodyne, cardiotonic, digestive, diuretic, cough dyspepsia, hemorrhoids, hepatosplenomegaly, laxative, stimulant, stomachic, uterine tonic, intermittent fever, jaundice, and leucoderma. The root bark is useful as an emetic, expectorant, and analgesic for bodyache and root juice in the treatment of snakebite [91]. The plant extract is also useful in the treatment of piles, colic pain, dropsy, phlegm, eye troubles, cardiac, and respiratory diseases.

## 8. Bioavailability and Toxicity

Bioavailability is a key issue in terms of effectiveness of any herbal medicine as a drug and will determine its effective delivery into the circulatory system in the body. Bioavailability of gymnemic acid is an important parameter for its *in vivo* pharmacological applications. Gymnemic acid has poor lipid solubility and complex structure and difficult to pass through the biomembranes for its absorption in circulatory system. Pathan and coworkers have developed a herbal formulation (gymnemic acid: phospholipid complex) with an aim to improve its bioabsorption and pharmacokinetics. A phytosome exhibits better absorption and utilization in body due to its increased capacity to cross lipid biomembranes and reach the systemic circulation. The complex exhibits antiapoptotic potential in doxorubicin-induced cardiotoxicity in rats and shows cardioprotective effect [92]. Toxicity studies of *Gymnema sylvestre* extract have shown its safety when taken in recommended doses. High doses may lead to side effects including hypoglycemia, weakness, shakiness, excessive sweating, and muscular dystropy. Administration of 1.00% basal powder (GSE) in the diet in Wistar rats for 52 weeks has shown no toxic effects and no animal died during the experiment [93]. Treatment of diabetic patients with *Gymnema sylvestre* has been shown to cause toxic hepatitis or drug-induced liver injury (DILI) [94].

## 9. *In Vitro* Cultivation of *Gymnema sylvestre*

Cultured plant cells and tissues are widely recognized as promising alternatives for the production of valuable secondary metabolites [95, 96]. Plant tissue culture techniques have been employed on an industrial scale for the production of bioactive compounds [97]. Various techniques were employed for propagation of the herb in plant tissue culture through *in vitro* multiplication for shoot regeneration from mature nodal explants of *G. sylvestre* [98] and large-scale production of gymnemic acids in plant cell suspension cultures [99]. Somatic embryogenesis was optimized and whole plant regeneration was achieved in callus cultures derived from hypocotyl, cotyledon, and leaf explants excised from seedlings of *G. sylvestre*. Globular/heart shaped embryos developed and produced torpedo and cotyledon stage embryos upon subculturing on embryo maturation medium EM8 (medium containing MS salts, B5 vitamins, 0.5 *μ*M BA, and 2% sucrose). The mature embryos were subcultured on fresh EM8 medium for embryo germination and plantlet formation. These plantlets were grown in glasshouse, respectively [100].

For *in vitro* regeneration of mature nodal explants of *G. sylvestre*, Murashige and Skoog (MS) media were used for the inoculation of single node explants having different combinations of 6-benzylaminopurine (BAP) or kinetin with naphthaleneacetic acid (NAA) and auxins like indoleacetic acid (IAA) alone or in combinations. The MS medium containing BAP (5 mg/L) and NAA (0.2 mg/L) exhibited maximum number of shoot (7 per explants). Further, the regenerated shoots were subjected to rooting on MS half strength medium in absence of any growth regulator (IAA, IBA, and NAA). In cultures where the shoot explants were inoculated on auxin-free half strength MS basal medium, root primordia emerged from the shoot base 15–20 days after implantation and subsequently developed into roots without basal callus as compared to MS media supplemented with different concentrations of auxins, which did not lead to root formation [15].

Plant cell suspension cultures were generated for large-scale production of gymnemic acids, the antisweet phytoconstituents. The methodology employed led to the development of a novel cell culture system for *in vitro* growth and cultivation of this species. The conditions for the production and HPLC quantification of gymnemic acids were optimized. The gymnemic acids were not accumulated in callus but were released into the medium. For the production of gymnemic acid commercially, this needs to be further optimized. In another study, the extraction of gymnemic acid through gymnemagenin from callus culture of *G. sylvestre* was reported. The aglycon component, known as gymnemagenin, was extracted, detected, and quantified in different callus cultures of *G. sylvestre*. HPTLC method was standardized for the rapid and accurate quantitative estimation of gymnemagenin in callus cultures of *G. sylvestre* [101].

Recently, Devi and Srinivasan [102] attempted the large-scale production of gymnemic acids under *in vitro *conditions, through the mediation of fungal elicitors. The use of bioelicitors, such as *Aspergillus niger* cell extract, enhanced the production of secondary metabolite, namely, gymnemic acids from *G. sylvestre *suspension culture. It is interesting to note that the elicitation of *Gymnema* suspension culture by *A. niger* significantly enhanced the production gymnemic acid as compared to nonelicited cultures. The technique is a potential means for the establishment of large-scale production of gymnemic acids through the employment of shaking flask and bioreactors. Due to the limited availability of *G. sylvestre* formulation, this technique holds good prospects for large-scale commercial production of bioactive phytoconstituents [102].

Gymnemic acid being an important bioactive compound, cell suspension cultures of *G. sylvestre* were generated and optimized for the production of gymnemic acids [103, 104].

## 10. Summary and Future Prospects

Medicinal plants served as a platform for ancient Ayurvedic system of medicine. In the present scenario, herbal therapeutics are gaining momentum in pharmacological applications and as molecular targets in the drug development. The emerging trend in rising incidence of diseases and associated complications with commercial medications poses a serious threat to mankind. Naturopathic treatments offer respite from the high cost of expensive drugs as well as in being comparatively safe with less side effects. It is estimated that nearly 80% of population depends on the natural remedies for health care. Plants are a valuable source of a number of bioactive compounds like alkaloids, quinine, paclitaxel, opium alkaloids, quinine, atropine, and cardiac glycosides (digitalis, ouabain) to name a few. The first antidiabetic drug, metformin, isolated from *Galega officinalis*, was a herbal formulation. Thus, it becomes very important to screen plants with pharmacological significance as a basis for the development of newer and more effective therapeutics. In spite of the good prospects of herbal medicines, these have gained little importance due to absence of scientific validation. The lack of availability of standards for herbal formulations is a major limitation. Although, a vast repertoire of plant resources is available but very few have experimentally validated and scientifically approved as medications for the treatment of diseases.

One major factor that comes into play is that many medicinal plants of commercial importance face threat of extinction due to increase in demand and destruction of their habitats due to urbanization and industrialization. The prime initiative should focus on the cultivation and conservation of medicinal plants with pharmacological importance. Although, the herb has immense prospects in drug development, but it faces threat of extinction due to continuous deforestation and absence of established lines or varieties. The *in vitro* propagation of plants, in plant tissue culture offers a promising alternative for the production of valuable secondary metabolite. *G. sylvestre, *being a valuable medicinal plant and source of bioactive substances, needs to be propagated and conserved. *In vitro* propagation of plants with high bioactive content and cell culture technologies for large-scale production of such secondary metabolites with medicinal significance will be highly prospective and will provide new dimensions to this area of research. Studies have been made in the past few years to understand the complex and incompletely understood nature of plant cells *in vitro* cultures [105]. Bioelicitors based strategies (from *Xanthomonas spp.* and *A. niger* cell extract) for enhanced production of gymnemic acids have been employed [102, 106], and the technique finds relevance for large-scale production of these bioactive compounds in bioreactors based industrial applications. These new technologies will be new beginning for further production and utilization of these sweet suppressing compounds invaluable as an antidiabetic herbal cure.

*G. sylvestre* holds a unique position among the sweetness modifying materials of natural origin. The herb accounts for multiple pharmacological significance as a naturopathic medication since ancient times and gaining popularity in the present scenario as well. Various polyherbal formulations like Dihar [53] and D-400 [105] containing *G. sylvestre* extract have been used for the treatment of diabetes mellitus. Several clinical trials and experimental studies indicated that the plant is an invaluable source of bioactive compounds and phytoconstituents like gymnemic acids have been used as molecular targets in drug development. Besides having pharmacological importance, the herbal extract exhibits good prospects in dietary applications. *G. sylvestre* dried leaf powder is orally consumed by Paliyan tribes of Sirumalai hills for treatment of diabetes. Several products such as GNC Herbal Plus Standardized *G. sylvestre* (herbal supplement), Vitamin Shoppe G. Sylvestre (sugar destroyer), Gymnema gold (Nutrigold) abolishe the taste of sugar and help support healthy glucose; Gurmar capsules (stimulates the heart and circulatory system and activate uterus) are some of the products composed of *Gymnema* extract and are marketed and sold as herbal preparations. Among the medicinal plants, *G. sylvestre* is a herb less exploited for its innumerable advantages. The aim of this review is to highlight the prospects of this rare herb as a potential medication for treatment of diseases from diabetes, obesity to cardiovascular disorders as well as a very good dietary and health supplements in food industry as an health tablets, beverages, tea bags, energy supplements, and in food items which regulates body weight. Gymnema sylvestre 75 is a herbal preparation which contains 75% Gymnemic acid from leaf extract and provides nutritional support to pancreas and maintain healthy blood sugar balance when used as part of diet.

The whole genome sequencing projects and functional elucidation of pathway genes have made significant contributions in deciphering the biological role and properties of biomolecules. With the functional characterization of genes, their relevance in the plant and functional role in the bioactivity of phytomolecules are being established. Information about such genes which code for economically viable traits or pharmacologically important bioactive molecules holds great prospects in crop engineering. The development of genetic transformation systems will provide an edge in the propagation and maintenance of such pharmacologically important plant having applications in drug discovery and development.

## Supplementary Material

Supplementary Table 1 describes the occurrence of gymnemic acid in various plant parts of *Gymnema sylvestre*. The highest percentage of gymnemic acid is present in shoot tip (54.29 mg g−1 DW) and lowest is in seeds (1.31 mg g^−1^ DW) respectively. Supplementary Table 2 summarizes the therapeutic potential of various plant parts and their application in pharmacological studies. Supplementary Table 3: Various bioactive phytoconstituents present in *Gymnema sylvestre*, their isolation and application in treatment of various health ailments.

## Figures and Tables

**Table 1:** Phytoconstituents in* Gymnema sylvestre*.