# Supplementary material for: Phytochemical and Pharmacological Properties of Gymnema sylvestre: An Important Medicinal Plant
Source: Biomed Res Int. 2014 Jan 6;2014:830285. doi: 10.1155/2014/830285 (PMC3912882; doi:10.1155/2014/830285)
Supplement: Supplementary file 1 — Supplementary Table 1 describes the occurrence of gymnemic acid in various plant parts of Gymnema sylvestre. The highest percentage of gymnemic acid is present in shoot tip (54.29 mg g−1 DW) and lowest is in seeds (1.31 mg g−1 DW) respectively. Supplementary Table 2 summarizes the therapeutic potential of various plant parts and their application in pharmacological studies. Supplementary Table 3: Various bioactive phytoconstituents present in Gymnema sylvestre, their isolation and application in treatment of various health ailments. [file 830285.f1.doc]

**Supplementary Table 1: Gymnemic acid content in various plant parts of *G. sylvestre* (taken from reference [8])**

| **Plant part** | **Amount of**  **gymnemic acids** |
| --- | --- |
| **Shoot tip** | 54.29 mg-g^-1^DW |
| **flowers** | 31.66 mg-g^-1^DW |
| **nodes** | 28.82 mg-g^-1^DW |
| **leaves** | 27.67 mg-g^-1^DW |
| **internodes** | 25.39 mg-g^-1^DW |
| **roots** | 20.56 mg-g^-1^DW |
| **seeds** | 1.31 mg-g^-1^DW |

**Supplementary Table 2: Plant parts of *G.sylvestre*** used as herbal therapeutics

| **Plant part used** | **Application** | **Referance** |
| --- | --- | --- |
| **Leaves** | Lowers blood sugar levels.  Prevention of dental plaque and caries.  Cardiovascular diseases.  As stimulant and diuretic | [12], [31], [62], [63], [65], [67] |
| **Leaf extract** | Antimicrobial activity  Anti-inflammatory effect | [68], [69], [70] |
| **Leaf powder** | Stimulates heart and the circulatory system, increases secretion of urine and activates uterus.  Reputed as a remedy for snake bite.  Used in stomachache. | [31], [71], [72] |
| **Root** | Expectorant and emetic.  Used in stomach pain.  Reputed as a remedy for snake bite. | [9] |
| **Root bark** | Treatment of piles | According the Bagbhat, rootbark useful in piles (Vedas) |
| **Root paste** | External application is useful in insect bites. | [73] |

**Supplementary Table 3: The compounds isolated from *G. sylvestre* and their bioactivity**

| **S.no.** | **Pharmacological application** | **Plant part** | **Isolated compound(s)** | **References** |
| --- | --- | --- | --- | --- |
| **1.** | Antidiabetic | Leaves | Gymnemic acids and corresponding glycosides | [26],[74] |
|  |  |  | Gymnemic acid 3 and Gymnemic acid 4 | [59] |
|  |  |  | Dihydroxy gymnemic triacetate | [75] |
|  |  |  | Gurmarin | [76] |
|  |  |  | Gymnestrogenin | [42] |
| **2.** | Anti-arthritic | Leaves | Leaf extract including tannins and saponins | [61] |
| **3.** | Anti-caries | Leaves | Gymnemic acid | [77] |
| **4.** | Antimicrobial | Roots | Pure saponin fractions | [78] |
| **5.** | Anti-viral | Leaves | Gymnemic acid A, B, C and D  Gymnemagenol | [79]  [80] |
| **6.** | Anti-larvicidal | Leaves | Gymnemagenol | [81] |
| **7.** | Anti-parasitic | Leaves | Gymnemagenol | [82] |
| **8.** | Anti-inflammatory | Leaves | Aqueous extract of *Gymnema sylvestre* leaves | [69] |
| **9.** | Anticancer and cytotoxic | Leaves | Deacyl Gymnemic acid  Gymnemagenin  Gymnemagenol | [83]  [84] |
| **10.** | Antidote for snake venom | Roots | Gymnemic acid | [85] |
| **11.** | Antihyperlipidemic | Leaves | Gymnemic acid  Gymnemate | [86] |
| **12.** | Immunomodulatory | Leaves | Tannins | [109] |
| **13.** | Hepatoprotective | Leaves | Hydro- alcoholic extract | [87] |
| **14.** | Wound healing | Leaves | Hydro- alcoholic extract  ( Gymnemic acid I, II, III and IV) | [88] |
| **15.** | Antisweet | Leaves | Gurmarin  Gymnemic acid | [89] |
| **16.** | Anti-apoptotic | Leaves | Gymnemic acid-phospholipid complex | [90] |
